# A further insight into the biosorption mechanism of Pt(IV) by infrared spectrometry

**DOI:** 10.1186/1472-6750-9-62

**Published:** 2009-07-06

**Authors:** Zhongyu Lin, Ru Xue, Yiwen Ye, Jianhong Zheng, Zhenling Xu

**Affiliations:** 1 State Key Laboratory for Physical Chemistry of Solid Surface, Department of Chemistry, College of Chemistry and Chemical Engineering, Xiamen University, Xiamen 361005, PR China; 2 Hospital of Xiamen University, Xiamen 361005, PR China

## Abstract

**Background:**

Platinum nanomaterial is one of the significant noble metal catalysts, and the interaction of platinum with microbe is one of the key factors in influencing the size and the distribution of the platinum nanoparticles on the microbial biomass. Some properties of Pt(IV) adsorption and reduction by resting cells of *Bacillus megatherium *D01 biomass have once been investigated, still the mechanism active in the platinum biosorption remains to be seen and requires further elucidating.

**Result:**

A further insight into the biosorption mechanism of Pt(IV) onto resting cells of *Bacillus megatherium *D02 biomass on a molecular level has been obtained. The image of scanning electron microscopy (SEM) of the D02 biomass challenged with Pt(IV) displayed a clear distribution of bioreduced platinum particles with sizes of nanometer scale on the biomass. The state of Pt(IV) bioreduced to elemental Pt(0) examined via X-ray photoelectron spectroscopy (XPS) suggested that the biomass reduces the Pt(IV) to Pt(II) followed by a slower reduction to Pt(0). The analysis of glucose content in the hydrolysates of D02 biomass for different time intervals using ultraviolet-visible (UV-vis) spectrophotometry indicated that certain reducing sugars occur in the hydrolyzed biomass and that the hydrolysis of polysaccharides of the biomass is a rapid process. The infrared (IR) spectrometry on D02 biomass and that challenged with Pt(IV), and on glucose and that reacted with Pt(IV) demonstrated that the interaction of the biomass with Pt(IV) seems to be through oxygenous or nitrogenous chemical functional groups on the cell wall biopolymers; that the potential binding sites for Pt species include hydroxyl of saccharides, carboxylate anion and carboxyl of amino acid residues, peptide bond, etc.; and that the free monosaccharic group bearing hemiacetalic hydroxyl from the hydrolyzed biomass behaving as an electron donor, in situ reduces the Pt(IV) to Pt(0). And moreover, the binding of the Pt(IV) to the oxygen of the carbonyl group of peptide bond caused a change in the secondary structure of proteins; i.e. a transformation, in polypeptide chains, of β-folded to α-helical form; it might be expected to be more advantageous than β-folded form to the platinum nanoparticles under shelter from gathering although the both special conformations of proteins could be much probably responsible for the stabilization of the particles.

**Conclusion:**

That knowledge could serve as a guide in the researches for improving the preparation of highly dispersive supported platinum catalyst and for fabricating new advanced platinum nanostructured devices by biotechnological methods.

## Background

The roles the microorganisms play in biotechnological applications including effective recovery of noble metal ions [1-3], creation of nanoscale materials with advanced structures [4,5] and preparation of highly dispersive supported noble metal catalysts [6,7] have been followed with a growing recent interest. Platinum nanomaterial is one of the significant noble metal catalysts, and the interaction of the platinum with the microbe is one of the key factors in influencing the size and the distribution of the platinum nanoparticles on the microbial biomass. Some properties of the interaction of Pt(IV) with resting cells of *Bacillus megatherium *D01 biomass were described previously [8], and yet the biosorption mechanism involved is still disputable and requires further expounding. The aim of our current research is based on our previous studies to make a further investigation into the mechanism responsible for the platinum biosorption by resting cells of *Bacillus megatherium *D02 biomass using IR and other spectroscopic techniques.

The strain D02 was screened out from different bacterial strains that were isolated from soils and waters of mining areas, because it has a relatively strong ability to adsorb and reduce Au(III), Ag(I), Pt(IV), Pd(II) and Rh(III). The strain is Gram-positive and identified as *Bacillus megatherium *D02 and easy to obtain and culture. It was cultured in an aqueous solution containing beef gels, peptone, salt, etc. [9]. The adsorptive capacity of the resting cells of the strain for Pt(IV) attained 76.6 mg/g when the biomass suspension (pH 3.5, 1 mg/ml) mixed with a 0.5 mM chloroplatinic acid (H_2_PtCl_6_·6H_2_O) aqueous solution at pH 3.5 and 37°C for 2 hours (h).

The chloroplatinic acid in an aqueous solution is generally in the form of PtCl_6_^2- ^anion, i.e. six-coordinate, octahedral complex. While the contact with D02 biomass suspension at pH 3.5, the liberation of chloride ion from the platinum complex occurred and the rate of the release was essentially as fast as that of the adsorption of the metal [10]. The interaction is thought that the functional groups locating in the cell wall biopolymers were easy of protonation and positively charged at this pH condition, and could rapidly adsorb the PtCl_6_^2- ^anion due to electrostatic interactions. The process therefore is believed to involve the initial formation of an ion pair between negatively charged PtCl_6_^2- ^and positively charged oxygenous or nitrogenous functional groups on the biomass, followed by elimination of chloride [10]; thus the resultant adsorbate on cell walls should be the Pt(IV) cation and the remnant, i.e. the free platinum species remaining in the aqueous solution, is still the PtCl_6_^-2 ^anion at early stage. As the interaction of the D02 biomass with the platinum occurred mainly between the functional groups of the biomass and their adsorbate, i.e. the Pt(IV) cation, it therefore was described to replace the PtCl_6_^-2 ^in the text. Similarly Pt(II) for PtCl_4_^-2^.

The appearance of Pt(IV) challenging D02 biomass was observed by SEM examination; the reducing ratios of Pt(IV) to Pt(II) and Pt(II) to Pt(0) by the biomass were determined using XPS and the hydrolysates of the biomass for different time intervals were analyzed for glucose content via UV-vis spectrophotometry. The interaction of chemical functional groups from the cell wall surfaces of the biomass with Pt(IV) was further studied by means of IR spectroscopic technique.

## Methods

### Biosorbent preparation

The biosorbent was prepared after a reported method [9]. The harvest of the cultural cells of D02 biomass was controlled at the growth stage. The D02 biomass sample was obtained by centrifuging at 3500 rings per minute (r./min) for 10 min on a centrifuge, washing with deionized water to remove any soluble substances that could interfere with the Pt(IV) studied, and then drying under vacuum at 80°C for 3 days (d). The dried sample was ground into powder and the resulting fine powder was then stored in a desiccator for use.

### SEM and XPS examinations

SEM examination: Two samples of (I) blank D02 biomass powder and (II) that challenged with Pt(IV) at pH 3.5 and 37°C for 48 h followed by drying under vacuum at 80°C and then by grinding were sprayed with gold prior to examination. The specimens were then examined under a LEO-1530 scan electron microscope (Germany).

XPS examination: Two samples of the D02 biomass powder challenged with Pt(IV) at pH 3.5 and 37°C, respectively, for 12 and 24 h were dried under vacuum at 80°C to be pressed into pellets prior to examination. The specimens were then determined on a PHI Quantum 2000 X-ray photoelectron spectrometer (USA). The spectra were recorded by monochromatized Al *Kα *radiation with *hv *of 1 486.60 eV and using the binding energy of 284.7eV of the amorphous carbon C1s as a reference point.

### UV-vis spectrophotometry

Two 30 mg samples of the D02 biomass powder were suspended separately in 6 ml of diluted HCl with final biomass concentration of 5 mg/ml and the pH adjustment at 3.5. Two milliliters of the biomass suspension (10 mg biomass) was placed separately into 6 test tubes followed by centrifugation to remove the supernatants. A 2 ml of deionized water at pH 3.5 was added to each of the six samples of the biomass followed by shake at 130 r/min in an incubator at 37°C; two sections of three samples each for the respective reaction times of 10 min and 24 h were centrifuged at 3500 r./min for 8 min. The supernatant of each tube, i.e. the hydrolysate of the biomass, was analyzed for glucose content by a phenol-sulfuric acid method [11,12] at 488 nm using a 752 UV-visible spectrophotometer (China).

### IR spectrometry

Generally, biological samples contain much water that strongly interferes with IR absorptions; in order to get rid of the water disturbing, the samples must be dried as much as possible to thoroughly eliminate the liquid water prior to examination. Two samples of (I) the hydrolysate of D02 biomass on hydrolysis for 24 h, just from the above sample for analyzing the glucose content and (II) that challenged with Pt(IV) at pH 3.5 and 37°C for 2 h; and six samples of (III) D02 biomass powder (IV) that challenged with Pt(IV) at pH 3.5 and 37°C for 2 d (V) 4 d (VI) 6 d (VII) 8 d and (VIII) 10 d were analysed. Three other samples of (IX) chloroplatinic acid powder (X) anhydrous glucose powder and (XI) a close to saturated aqueous solution of chloroplatinic acid powder fully mixed with anhydrous glucose (1.4: 1 in gravimetric ratio) were heated respectively in an oil-bath at 100°C for 60 min followed by drying prior to examination. The samples for IR analysis were prepared by pressing powdered KBr pellets mixed intimately with about 5% – 10% of finely ground powder of the each sample, and then determined on a Nicolet 740SX FTIR spectrophotometer with a MCT-B detector (USA). The spectra were recorded in the range 4 000 ~ 625 cm^-1 ^at a resolution of 4 cm^-1 ^with 32 scans.

## Results and discussion

### SEM and XPS Characterizations of Pt(IV) biosorption

**Figure 1:**
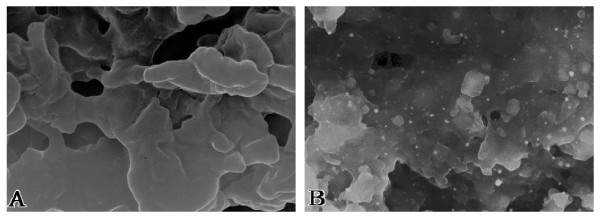
**SEM images of (A) *Bacillus megatherium *D02 biomass and (B) that challenged with Pt(IV) for 48 h**. (Mag = 30.00 K×).

Under the scan electron microscope, the blank D02 biomass hadn't any small metallic particles to be seen (Figure 1A), but that challenged with Pt(IV) showed a clear distribution of tiny, bright, bioreduced platinum particles with sizes of a few to more than a hundred nanometers (nm) on the biomass (Figure 1B). Further analysis of the specimens of the Pt(IV) challenging the biomass for different time intervals was preformed by XPS technique, which gave spectra with peaks of binding energy of 75.2, 74.7 and 71.7 eV corresponding, respectively, to Pt(IV) (4f, 7/2), Pt(II) (4f, 7/2) and Pt(0) (4f, 7/2). It was estimated at about 27.1% Pt(IV), 57.8% Pt(II) and 15.1% Pt(0) for 12 h; and 16% Pt(IV), 61.2% Pt(II) and 22.8% Pt(0) for 24 h by analyzing the peak areas. Thus, the reductive ratio of Pt(II) is 57.8%, Pt(0) 15.1% for 12 h and Pt(II) 61.2%, Pt(0) 22.8% for 24 h; indicating that the biosorption of the Pt(IV) by the biomass involves the reduction of Pt(IV) to Pt(II) followed by a slower reduction to Pt(0). The both examinations reflected that the bioreduction of Pt(IV) to elemental Pt(0) at near normal temperature was evidently catalyzed by D02 biomass and that certain enzymes originating from the biomass could be much responsible for this catalysis. The result suggests that the biomass must have served as a catalyst as well as a role in sheltering the platinum nanoparticles from gathering for helping the stabilization of the particles to a certain extent besides as an electron donor in the Pt(IV) bioreduction.

### Analysis for glucose content in D02 biomass

**Figure 2:**
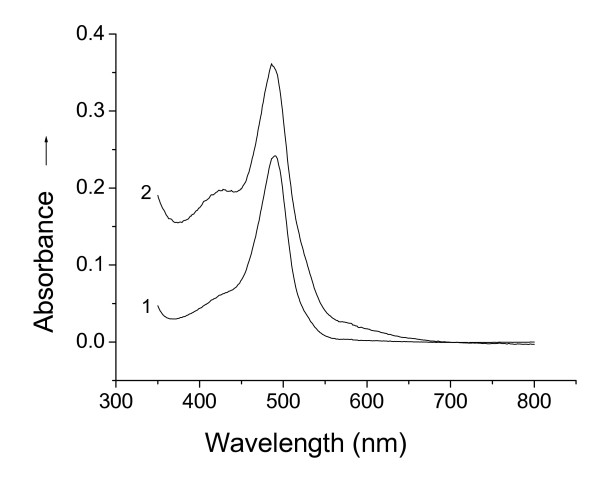
**UV-vis spectra of hydrolysates of *Bacillus megatherium *D02 biomass on hydrolysis for (1) 10 min and (2) 24 h**.

The UV-vis spectra of the hydrolysates of D02 biomass on hydrolysis for 10 min and 24 h respectively displayed an absorption band near 488 nm arising from glucose (Figure 2). It showed that the glucose content in the hydrolysate corresponded to 2.52% of the biomass dry weight on hydrolysis for 10 min (curve 1), and to 3.78% of that for 24 h (curve 2). As a general rule, the hydrolysate of the biomass also contains other reducing sugars including oligosaccharides, dioses, monoses, etc. besides the glucose, so the amount of the total reducing sugars in the hydrolyzed biomass must be far larger than 2.52% and 3.78%, respectively, for 10 min and 24 h. The glucose content on hydrolysis for only 10 min had already approached 67% of that for 24 h, showing that the hydrolysis of the polysaccharides of the biomass is a rapid process and may limit on the cell wall surfaces. This behavior of the biomass provided a favorable condition for the following Pt(IV) bioreduction and then the formation of the platinum nanoparticles.

### IR Characterization of Pt(IV) biosorption

**Figure 3:**
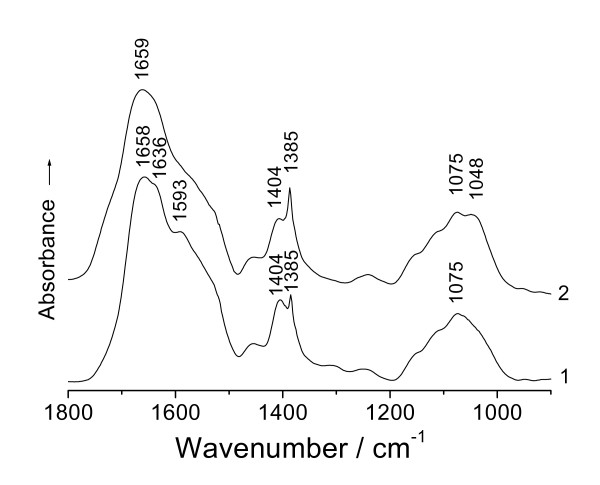
**FTIR spectra of (1) hydrolysate of *Bacillus megatherium *D02 biomass on hydrolysis for 24 h and (2) that challenged with Pt(IV) at pH 3.5 for 2 h**.

For better understanding of the action of the platinum on both carboxylate anion group (O = C-O^-^) and free hydroxyl (C-O-H) of the cell walls, an IR comparative study between the hydrolysate of D02 biomass and that challenged with Pt(IV) was performed. The use of the hydrolysate in this case would avoid any insoluble impurities that could interfere with the IR spectra studied. The spectrum of the hydrolysate displayed absorptions near 1 593 and 1 404 cm^-1 ^(Figure 3, curve 1), respectively, assigned to asymmetric and symmetric  stretching bands of the carboxylate anion group [13,14]. After the contact with Pt(IV) at pH 3.5, the protons came into existence in the system; the hydrolysate exhibited a spectrum with clear changes at 1 593 and 1 404 cm^-1 ^due to the complexation of the carboxylate anion group by coordination with Pt(IV) [15]. The binding of Pt(IV) to the oxygen of the carboxylate resulted respectively in a blue (higher frequency) shift of the asymmetric  absorption as well as a red (lower frequency) shift of the symmetric band [15], which could be correlated with covalent bond formation between Pt(IV) and oxygen; thus the disappearance of the asymmetric  band near 1 593 cm^-1 ^and a decrease in the intensity of the symmetric at 1 404 cm^-1 ^(Figure 3, curve 2). Here must be pointed out that the carbonyl absorption of carboxyl (O = C-OH), a most sensitive to IR absorption, should be at about 1726 cm^-1 ^if occurring; but it wasn't found in Figure 3, curve 2 after the hydrolysate in contact with the protons. The reason for the absence of the carboxyl absorption is likely that the carboxylate anion of the hydrolysate reacted with the protons was through electrostatic attractions and still retained its ionic character. Thus, there was naturally no the carbonyl absorption of the carboxyl in IR. This just indicates that the action of the protons on the carboxylate anion is only ionic interaction and the protons being in the system cannot possibly influence the IR absorption of carboxylate anion or carboxyl in this case. The result serves as an important basis for the following discussion about the mechanism of the redox reaction of the biomass with Pt(IV). Another change can be observed on the appearance of one new band at 1 048 cm^-1 ^besides the other at 1 075 cm^-1 ^(Figure 3, curve 2), due to the interaction of the oxygen of the hydroxyl from saccharides with the platinum [13], which led to a red shift of the band from 1 075 to 1 048 cm^-1^. The cases of metal uptake and binding by both carboxylate anion group and hydroxyl from the cell wall tissues of the biomass have also been found in *Saccharomyces cerevisiae *[2], *Lactobacillus *sp. strain A09 [3], *Bacillus licheniformis *R08 [16] and so forth.

**Figure 4:**
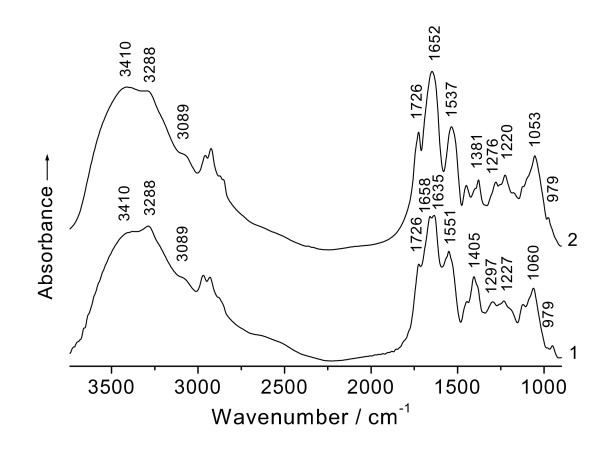
**FTIR spectra of (1) *Bacillus megatherium *D02 biomass and (2) that challenged with Pt(IV) at pH 3.5 for 48 h**.

The IR spectrum of D02 biomass displayed an absorption at 1 551 cm^-1 ^corresponding to the δ_N-H _+ ∪_C-N_, a coupled vibration including N-H in-plane bending and C-N stretching modes of the amideIIband originating from the C-N-H group of the peptide bond (HNC = O) [13,17] (Figure 4, curve 1). After the contact with Pt(IV) for 48 h, there had been Pt(II) and Pt(0) besides Pt(IV) in this system and the biomass exhibited the spectrum with a clear shift of the δ_N-H _+ ∪_C-N _from 1 551 to 1 537 cm^-1 ^(Figure 4, curve 2), due to the binding of Pt species to the nitrogen of the peptide bond; a similar aspect for the same reason can be observed in the situation of the amide III band (major contribution from a mixed vibration involving C-N and N-H modes), in which two shifts occurred from 1297 and 1227 cm^-1 ^to 1276 and to 1220 cm^-1 ^[13] respectively (Figure 4, curve 2). Another decrease in the intensity of the ∪_N-H _band of the bonded N-H from the peptide bond can be observed at 3 288 cm^-1 ^[13], which is also because of the binding of the platinum to the nitrogen. One shoulder peak near 3 089 cm^-1 ^originating in an overtone of the amideIIband was also associated with the N-H mode [13], its intensity showed nearly unchanged rather than obviously decreased and finally missing as prolonging the reaction time [8]; so the band can be neglected in this case.

As indicated earlier, two clear increases in the intensities of absorptions of the saccharide hydroxyl at 3 410 cm^-1 ^(∪_O-H_) and 1 053 cm^-1 ^(δ_O-H _+ ∪_C-O_) can be found in Figure 4, curve 2. The reason in chief resulted from an increase in quantity of the free hydroxyl, namely, the hydrolysis of some polysaccharides to shorter saccharides [2,16] such as oligosaccharides, dioses, monoses, etc.; most of which have the free monose group bearing the hemiacetalic hydroxyl and are of the reducing property like glucose and are referred to generally as reducing sugar. This further supported the existence of certain reducing sugars in this system. Therefore, one of the principal reasons for the two other intensifications of the carboxyl absorptions at 1 726 cm^-1 ^(∪_C = O_) and 979 cm^-1 ^(δ_O-H_) in IR must have resulted from the oxidation of reducing sugars to their corresponding acids by platinum cations [2,16].

**Figure 5:**
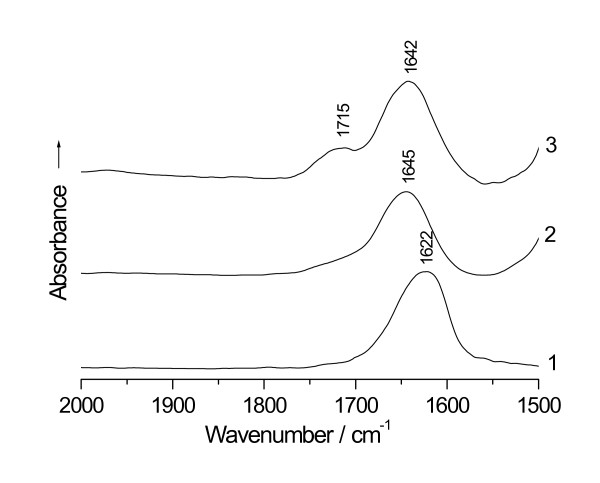
**FTIR spectra of (1) chloroplatinic acid (2) glucose and (3) glucose reacted with chloroplatinic acid after being heated respectively in an oil-bath at 100°C for 60 min**.



In order to verify the above inference; glucose, the commonest reducing sugar, was examined by IR for the interaction with Pt(IV). To be in comparison with each other; IR spectra of chloroplatinic acid, glucose and that challenged with Pt(IV) were shown in Figures 5, curve 1–3 respectively. In the 2 000 ~ 1 500 cm^-1 ^range, it can be observed that the spectra of chloroplatinic acid and glucose showed only a single band of H_2_O at 1 622 and 1 645 cm^-1 ^respectively, and the glucose reacted with Pt(IV) displayed a spectrum with the occurrence of the ∪_C = O _of the carboxyl at 1 715 cm^-1 ^besides one absorption of H_2_O at 1 642 cm^-1^. The result meant that the free aldehyde group shifted from the cyclic hemiacetalic hydroxyl of the glucose had already been oxidized to the carboxyl by the platinum cation. Further analysis of this sample using X-ray powder diffractometry gave a pattern with peaks corresponding exactly to those of the elemental Pt(0), which proved that the Pt(IV) had been reduced to the Pt(0) by the glucose under the reaction conditions. The redox reaction of the Pt(IV) with the glucose can be expressed as follows:

As a matter of fact, this is a model reaction of the Pt(IV) with the D02 biomass, the mechanism of the bioreduction of Pt(IV) to Pt(0) by the biomass can be assumed to be the same as that by the glucose. The biomass fulfilled the roles as a catalyst as well as an electron donor in this redox reaction. Both UV-vis and IR analyses testified that in the system some polysaccharides of the biomass had been hydrolyzed to reducing sugars; so when they met the Pt(IV) adsorbed on the cell wall surfaces, the reduction of Pt(IV) to Pt(II) followed by a slower reduction to Pt(0) occurred:



The free aldehyde group shifted from the cyclic hemiacetalic hydroxyl of various reducing sugars was oxidized to the carboxyl along with the Pt(IV) being reduced, hence two clear increases in the respective intensities of the carboxyl absorptions at 1 726 and 979 cm^-1 ^(Figure 4, curve 2). This similar microcosmic process of the metal bioreduction has also been found in some cases of Au^3+ ^in *Saccharomyces cerevisiae *[2], Ag^+ ^in *Lactobacillus *sp. strain A09 [3], Pd^2+ ^in *Bacillus licheniformis *R08 [16], etc. It is very probable that the analogous mechanism would be responsible to different kinds of various microbes for the bioreduction of the noble metals.

It has been reported that the carbonyl absorption of the carboxyl at 1726 cm^-1 ^disappeared after the contact with Pt(IV) [8], this result seems to be in contradiction with the mention made in just the above paragraph and requires further elaborating. The intensities of both ∪_C = O _(1 726 cm^-1^) and δ_O-H _(979 cm^-1^) from the carboxyl absorptions were found to change with prolonging the exposure time of the D02 biomass to Pt(IV). The ratios of the intensity of the ∪_C = O _of the carboxyl at 1 726 cm^-1 ^to that of the ∪_C = O _(the amideIband) of the peptide bond at 1 652 cm^-1 ^(i.e. I_1726_/I_1652_, a method for the semi-quantitative assessment of the carboxyl; because the quantity of the carbonyl of the peptide bond is far larger than that of the carboxyl, from the statistical viewpoint, the carbonyl absorption of the peptide bond can be generally regarded as an almost changelessness in its absorption intensity while binding the Pt species) for different time intervals are shown: 0.637 (2 d), 0.706 (4 d), 0.739 (6 d), 0.581 (8 d) and 0.423 (10 d). As seen from the values, the carbonyl absorption of the carboxyl becomes the most intense as the biomass challenged Pt(IV) for 6 days, and it becomes lower for 8 days and lowest for 10 days. Fourest et al. [18] noted that the protonated *Sargassum *biomass showed the IR spectra with an obvious decrease in the intensity of the free ∪_C = O _of the carboxyl at 1738 cm^-1 ^after the contact with Cd(II), the absorption became weak and finally disappeared with increasing the concentration of Cd(II). The result reflected that the carboxyl is also an active group for binding Cd(II) and there is not the occurrence of the redox reaction but only the binding action between *Sargassum *biomass and Cd(II). Based on electronegativity and steric effect points of view, the carboxyl could successfully compete with the peptide bond for binding Pt species [3]; but, after all, it is amino acid residues and a small quantity as compared with the peptide bond, so that the amido linkage still had the chance to bind Pt species anyway from the viewpoint of statistics. The above IR spectrum (Figure 3, curve 2) showed that in the present system the existence of protons cannot interfere in the carboxyl absorption; so we can generally infer that when the rate of both Pt(IV) and Pt(II) bioreduction was more rapidly than that binding to the carboxyl, the carbonyl absorption of the carboxyl must have tended to a progressive intensification (as the above, this process lasted 6 days). While 6 days later, as the bioreducing rate was slower than the binding to the carbonyl; or as the redox reaction was close to equilibrium, the carboxyl wouldn't be yielded any longer and it was going on binding Pt species [8]; both cases must have resulted in a red shift of the carbonyl absorption at 1726 cm^-1^, which caused a decrease in the intensity of this band.

It is of interest to note that the amideIband, due to the carbonyl stretching absorption of polypeptides, can be found to split into two peaks at 1 658 and 1 635 cm^-1 ^(Figure 4, curve 1), arising respectively from conformations of α-helical and β-folded in proteins [17], characterized largely by the respective periodic arrays of intra- and inter-molecular hydrogen bonds in polypeptide chains [19]. After the contact with Pt(IV), the biomass exhibited the spectrum with clear shifts of the ∪_C = O _from the both 1 658 and 1 635 cm^-1 ^to 1 652 cm^-1 ^(Figure 4, curve 2), which is attributed to the interaction of the carbonyl of the peptide bond with the platinum [17]. The shifts from the both to 1 652 cm^-1 ^suggested that the action of Pt species on the oxygen of the carbonyl of polypeptides must have resulted in a rupture of the inter-chain (i.e. inter-molecular) hydrogen bond linking neighboring peptide chains to destroy the original pleated structure of β-folded and led to the occurrence of α-helical conformation that has the regular arrays of intra-chain (i.e. intra-molecular) hydrogen bond. So a change in the secondary structures of proteins, namely, a transformation of β-folded to α-helical conformation, took placed. In general; α-helical form, the most content and the commonest secondary structure in proteins, bearing 169 atoms of both carbon and nitrogen in each of the helical rings closed by intra-molecular hydrogen bonding, has a fixed nanometer diameter and a pitch of 0.54 nm between the helices [20]. And β-folded, the second most quantity in the secondary structures of proteins, has the form of pleated sheet with regularity. At the initial rapid adsorption stage; most of the free PtCl_6_^-2 ^anion, i.e. an octahedral coordinated complex with diameter of about 0.65 nm, was quickly attracted on the surface of the proteins of the biomass and the release of chloride ion from the platinum complex took place simultaneously with the adsorption of PtCl_6_^-2 ^[10], so that the resultant adsorbate on the cell walls was just the Pt(IV) cation with diameter of about 0.248 nm. In the meantime, a transformation of β-folded to α-helical conformation was occurring; and the Pt(IV) cation bound by the oxygen of the carbonyl of the β-folded form on polypeptides might easy be carried through the pitches into the helical circles along with the change in the secondary structure of proteins. While the proteins with folded and helical conformations of polypeptide chains could much probably contribute to the stabilization of the platinum nanoparticles; from the structural point of view, α-helical form might be expected to be more advantageous than β-folded to the particles under shelter from gathering. If the best part of the Pt species could have been bound continuously to the chemical functional groups on the cell walls of the biomass or, the better, into the respective helices of polypeptides, or the pores of net-like structural polysaccharides [1], etc.; we could have obtained very homogenous platinum nanoparticles. However, the biosorption of the metal is highly pH dependence with the maximum adsorptive capacity near pH 3.5. When pHs < 3.5, most of the protons were able to compete with Pt(0), Pt(II) and Pt(IV) for the binding sides of active groups on cell walls, so that the adsorptive capacity of the biomass for Pt species decreased with the pH falling; when pHs > 3.5, it caused the precipitation of platinum hydroxides, which could also disturb the adsorption and led to reduce the adsorptive capacity. Actually, rather part of uneven nanoparticles can be found on the biomass (Figure 1B). One of the primary reasons for this is likely due to a decrease in pH of the biosorption system because both processes of Pt(IV) bound and reduced by the biomass usually cause the liberation of protons [3,16], thus resulting in further acidification of the present system. The drop in pH value from 3.5 to 2.5 occurred in a matter of 1 h after the biosorption, this being equal to a ten times the initial concentration of protons; and it was going on falling following the biosorption proceeding. In this case, the excessive protons could capture the binding sites of active groups from Pt species; which would cause rather part of Pt(0) to be separated from the biomass and no longer sheltered by the biological macromolecules. Then, the free nanoparticles would likely gather each other. To avoid the drop of Pt species from the biomass, the pH adjustment may have to be made in good time and the value has to be maintained at pH 3.5 during the biosorption process. Thus, there is hope of attaining more homogenous platinum nanoparticles; the work improving the size of the metallic nanoparticles remains to be done still further.

## Conclusion

The biosorption mechanism of Pt(IV) onto *Bacillus megatherium *D02 biomass on a molecular level has further been investigated mainly by infrared spectrometry in this report. The findings of the Pt(IV) bioreduced by the biomass to elemental Pt(0) at near normal temperature followed by the formation of platinum nanoparticles show that the biomass must have behaved as a catalyst as well as a role in sheltering the particles from gathering besides as an electron donor in this redox reaction. Further analysis suggests that the binding of the Pt(IV) to proteins led to a change, in polypeptide chains, of β-folded to α-helical form in that the latter might be expected to be more advantageous than the former to the nanoparticles under shelter from gathering, although the both special secondary structures of proteins could be much probably responsible for the stabilization of the particles. At all events, both patterns of the secondary constructions of proteins (e. g. α-helical, β-folded, etc.) and pores of the net-like structural polysaccharides on peptidoglycan layers of cell walls of the biomass may perform a significant function for stabilization and uniformity of the particles by having the pH under control in process of the biosorption. A better understanding of the biosorbent mechanisms responsible for Pt(IV) binding and reduction could contribute to the development of a method for fabrication of platinum nanoscale devices, and for an improvement of preparation of highly dispersive supported platinum catalysts by biotechnological methods.

## Authors' contributions

RX carried out the SEM examination, participated in the interpretation of the SEM images. YY carried out the XRD examination, participated in analysis and interpretation of XRD patterns. JZ and ZX carried out biosorption examinations, participated in the analyses of both adsorptive efficiency and capacity of the biomass. ZL conceived of the study and carried out the IR examinations and performed further synthetic analyses and drafted the manuscript. All the authors read and approved the final manuscript.
